# Photo Quiz: Annular penoscrotal papuloplaques with atypical targetoid macules

**DOI:** 10.1128/jcm.01598-25

**Published:** 2026-04-21

**Authors:** Xiao-Hua Li, Dan-Qiong Zheng, Yi-Ming Fan

**Affiliations:** 1 Dermatology, Plastic and Cosmetic Surgery Center, the First Dongguan Affiliated Hospital of Guangdong Medical University, Dongguan, Guangdong, China; Mayo Clinic Minnesota, Rochester, Minnesota, USA

## PHOTO QUIZ 

**Fig 1:**
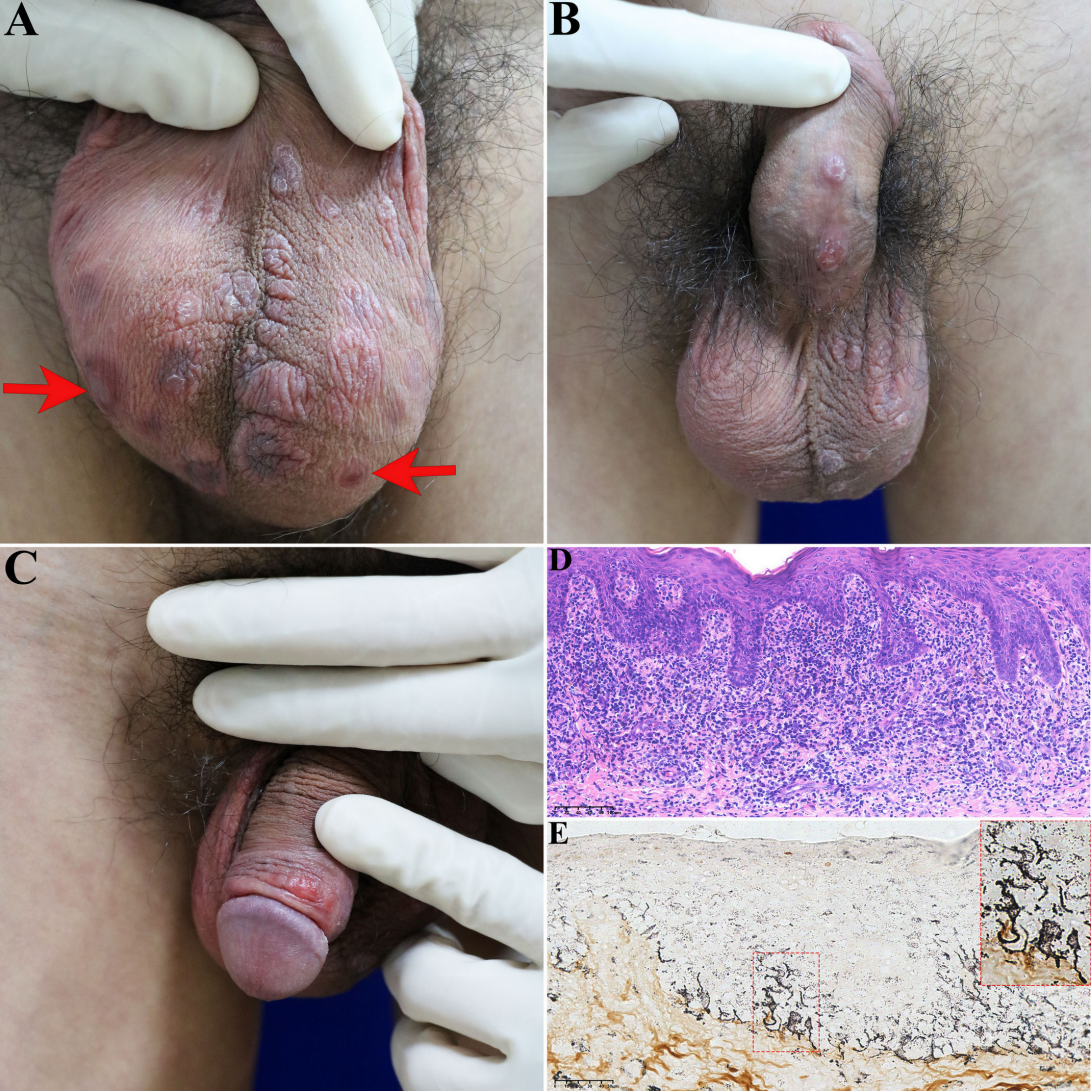
Clinical and histopathologic observation. (**A–C**) Multiple, discrete, annular, scaly, erythematous papules and plaques, and several atypical targetoid macules (arrows) on the scrotum (**A**) and ventral prepuce (**B**), and an edematous erythema with mild erosion on the dorsal foreskin near the coronal sulcus (**C**). (**D**) Hematoxylin-eosin stain showed irregular epidermal acanthosis and dermal lymphocytic and plasmacytic infiltration with vascular proliferation and endothelial swelling (original magnification ×200). (**E**) Warthin-Starry stain displayed multiple helical and thread-like spirochetes in the lower epidermis. The inset highlighted the spirochetes (original magnification ×400).

A 29-year-old unmarried man presented with a 1-month history of slightly pruritic erythematous eruptions on the scrotum in May 2025, which was presumptively diagnosed as “scrotal eczema” and was unresponsive to topical unidentified ointments in local hospital. Meanwhile, similar lesions occurred on the penis in the past 5 days. He recalled an unprotected sexual exposure with a heterosexual partner in November 2024 and developed painless erythema and erosion over the glans penis 1.5 months thereafter. The balanic lesion was initially diagnosed as “erosive balanitis” and healed 1 month after oral unidentified antibiotics and topical agents. Cutaneous examination revealed multiple, 4–15 mm in diameter, discrete, annular, scaly, erythematous papules and plaques over the scrotum and ventral foreskin of the penis, and several atypical targetoid/iris macules on the scrotum (Fig. 1A and B). An edematous erythema with mild erosion was noted on the dorsal foreskin near the coronal sulcus (Fig. 1C). There were no other mucocutaneous lesions, inguinal lymphadenopathy, and constitutional symptoms. Toluidine red unheated serum test (TRUST; 1:64 titer; Rongsheng Biotech, Shanghai, China) and *Treponema pallidum* particle agglutination (TPPA; Livzon Diagnostics Inc, Zhuhai, China) were positive, whereas HIV-1 antigen and HIV-1/HIV-2 antibodies (Sysmex Corporation, Kobe, Japan) were negative. Preputial biopsy showed irregular epidermal acanthosis and dermal lymphocytic and plasmacytic infiltration with vascular proliferation and endothelial swelling (Fig. 1D). Warthin-Starry stain displayed multiple helical and thread-like spirochetes in the epidermis (Fig. 1E).

## ANSWER TO PHOTO QUIZ

The presence of annular and atypical targetoid lesions on the penoscrotal area with a previous history of unprotected sexual exposure and “erosive balanitis” suggested the diagnosis of secondary syphilis, which was confirmed by reactive serologic testing (TRUST and TPPA) and Warthin-Starry stain. He was treated with weekly intramuscular benzathine penicillin G (BPG; 2.4 million units) for 2 weeks. The penoscrotal lesions resolved 3 weeks post-treatment, and serum TRUST titer decreased from 1:64 to 1:8 at the 4-month follow-up.

Secondary syphilis typically presents as generalized non-pruritic maculopapular rashes on the trunk and extremities and often involves the palmoplantar area (1, 2). However, atypical eruptions are reported in 25.8%–31% of cases, including psoriasiform, annular (ring-shaped), lichenoid, pustular, frambesiform, and nodular (2, 3).

The exclusive penoscrotal involvement with annular and atypical targetoid/iris (bullseye) rashes in this case is extraordinarily unusual. Annular syphilis manifests as slightly scaling papules or verrucous plaques and often affects the oral commissures, cheeks, scalp, palms, soles, and intertriginous areas but rarely the genital area (2–4). Main differential diagnoses include other annular lesions such as granuloma annulare, annular lichen planus, annular psoriasis, scabies, and dermatophytosis (4). Erythema multiforme-like eruption is extremely rare in secondary syphilis (5). Unlike classic targetoid/iris lesions consisting of three concentric segments (dark center and peripheral pinkish ring surrounded by a red halo), atypical lesions had two color zones in this case.

The diagnosis of secondary syphilis is made by the positivity of both treponemal (TPPA and *T. pallidum* hemagglutination) and nontreponemal (TRUST and rapid plasma reagin) tests. Nontreponemal tests are useful to monitor treatment response, relapse, and reinfection, while treponemal tests are generally more sensitive during early syphilis (1, 6). As with clinical manifestations, histopathologic findings of syphilis are highly variable. However, endothelial cell swelling/proliferation and dermal lymphocytic/plasmocytic infiltration are suggestive of syphilis (6). Although Warthin-Starry stain is hard to interpret due to low specificity and sensitivity (6), it is widely used in China. Additionally, immunohistochemical stain is sensitive and specific for detecting *T. pallidum* in secondary syphilis (6).

The first-line regimen for early syphilis (primary, secondary, or early latent syphilis) is 1 dose of intramuscular BPG in the US and European guidelines (1, 6) and 1–2 doses in China.

In conclusion, secondary syphilis should be included in the differential diagnosis of unusual penoscrotal lesions.

## References

[1] Janier M, Unemo M, Dupin N, Tiplica GS, Potočnik M, Patel R. 2021. 2020 European guideline on the management of syphilis. J Eur Acad Dermatol Venereol 35:574–588. doi:10.1111/jdv.16946 33094521

[2] Forrestel AK, Kovarik CL, Katz KA. 2020. Sexually acquired syphilis: historical aspects, microbiology, epidemiology, and clinical manifestations. J Am Acad Dermatol 82:1–14. doi:10.1016/j.jaad.2019.02.073 30986477

[3] Ciccarese G, Facciorusso A, Mastrolonardo M, Herzum A, Parodi A, Drago F. 2024. Atypical manifestations of syphilis: a 10-year retrospective study. J Clin Med 13:1603. doi:10.3390/jcm13061603 38541829 PMC10971508

[4] Montero-Menárguez J, Puerta-Peña M, Agud-de Dios M, Guzmán-Pérez LM, Gutiérrez-Collar C, Fulgencio-Barbarin J. 2023. Annular genital lesions, not an unusual presentation of secondary syphilis. J Dtsch Dermatol Ges 21:907–909. doi:10.1111/ddg.15086 37212344

[5] Liu H, Goh BT, Huang T, Liu Y, Xue R, Ke W, Gu M, Yang B. 2019. Secondary syphilis presenting as erythema multiforme in a HIV-positive homosexual man: a case report and literature review. Int J STD AIDS 30:304–309. doi:10.1177/0956462418805197 30482099

[6] Forrestel AK, Kovarik CL, Katz KA. 2020. Sexually acquired syphilis: laboratory diagnosis, management, and prevention. J Am Acad Dermatol 82:17–28. doi:10.1016/j.jaad.2019.02.074 30986474

